# Hexagonal Voronoi pattern detected in the microstructural design of the echinoid skeleton

**DOI:** 10.1098/rsif.2022.0226

**Published:** 2022-08-10

**Authors:** Valentina Perricone, Tobias B. Grun, Francesco Rendina, Francesco Marmo, Maria Daniela Candia Carnevali, Michal Kowalewski, Angelo Facchini, Mario De Stefano, Luigia Santella, Carla Langella, Alessandra Micheletti

**Affiliations:** ^1^ Department of Engineering, University of Campania Luigi Vanvitelli, Via Roma 29, Aversa 81031, Italy; ^2^ Division of Invertebrate Paleontology, Florida Museum of Natural History, University of Florida, Gainesville, FL 32618, USA; ^3^ Department of Science and Technology, University of Naples ‘Parthenope’, URL CoNISMa, Centro Direzionale Is.4, Naples 80143, Italy; ^4^ Department of Structures for Engineering and Architecture, University of Naples Federico II, Via Claudio 21, Naples 80125, Italy; ^5^ Department of Environmental Science and Policy, University of Milano, Via Celoria 26, Milan 20133, Italy; ^6^ IMT school for advanced studies Lucca, Piazza S. Ponziano 6, 55100, Lucca, Italy; ^7^ Department of Environmental, Biological and Pharmaceutical Science and Technology University of Campania ‘L. Vanvitelli’, Via Vivaldi 43, Caserta 80127, Italy; ^8^ Department of Research Infrastructures for Marine Biological Resources, Stazione Zoologica Anton Dohrn, Villa Comunale 1, Naples 80121, Italy; ^9^ Department of Architecture and Industrial Design, University of Campania Luigi Vanvitelli, Via San Lorenzo, 81031, Aversa, Italy

**Keywords:** echinoids, stereom, trabecular system, geometric pattern, Voronoi

## Abstract

Repeated polygonal patterns are pervasive in natural forms and structures. These patterns provide inherent structural stability while optimizing strength-per-weight and minimizing construction costs. In echinoids (sea urchins), a visible regularity can be found in the endoskeleton, consisting of a lightweight and resistant micro-trabecular meshwork (stereom). This foam-like structure follows an intrinsic geometrical pattern that has never been investigated. This study aims to analyse and describe it by focusing on the boss of tubercles—spine attachment sites subject to strong mechanical stresses—in the common sea urchin *Paracentrotus lividus*. The boss microstructure was identified as a Voronoi construction characterized by 82% concordance to the computed Voronoi models, a prevalence of hexagonal polygons, and a regularly organized seed distribution. This pattern is interpreted as an evolutionary solution for the construction of the echinoid skeleton using a lightweight microstructural design that optimizes the trabecular arrangement, maximizes the structural strength and minimizes the metabolic costs of secreting calcitic stereom. Hence, this identification is particularly valuable to improve the understanding of the mechanical function of the stereom as well as to effectively model and reconstruct similar structures in view of future applications in biomimetic technologies and designs.

## 1. Introduction

Regular geometric patterns pervade the natural world. As stated by Galileo Galilei in the Assayer (1623): ‘the book of nature is written in the language of mathematics'. Repeated arrays of polygons or polyhedra recur in biological (e.g. retinal cells and epithelia) and physico-chemical (e.g. crystalline structure, crack pattern, soap bubbles) systems [1–5]. In organisms, the emergence of a geometric order can correspond to adaptive functional traits such as the repeated pattern of triangles and hexagons. These geometries are the result of an evolutionary optimization process toward structural strength, minimized constructional energy, and space-filling maximization [1,4,6].

Many biological constructions can be specifically described as Voronoi models. A Voronoi model is a geometrical partitioning of two-dimensional or three-dimensional spaces into polygonal regions, known as Voronoi cells. These cells are generated around a pre-existing number of points, called seeds [7]. Voronoi models follow the nearest-neighbour rule, where all the points belonging to a given Voronoi cell are closer to its corresponding seed than to any other seed [8]. The boundary between two Voronoi cells is equidistant to the two nearest seed points [5]. Voronoi structures have been reconstructed and analysed in numerous studies with specific reference to their mechanical properties. The structural stability is mainly influenced by its regularity and geometrical characteristics, i.e. distribution, number, and shape of cells as well as cell wall thickness [9–17]. Polygonal Voronoi structures can vary from highly irregular to fully ordered constructions as from galaxy and molecule spatial distribution, oyster shell prismatic layer to the beeswax honeycomb [12,13,18–20]. These structures generally correspond to lightweight and load-bearing constructions with optimized stress transferring system [21–23]. A mathematical assessment and modelling of Voronoi structures facilitate the evaluation of bio-structural features, growth processes and structural-mechanical responses, as well as their effective reconstruction. Voronoi based structures can be used as high-performance solutions for material science, industrial products and building constructions [15,21,24–28]. There are numerous examples of Voronoi structures in nature, which include insect wing venation [29], turtle shell, trabecular bone [21], oyster shell prismatic layer [20], honeycomb, the cell wall of wood [21,30,31], the cell wall of coenobial green alga (*Pediastrum boryanum*) [32], chick embryo retinal pigment cells, lung epithelial cells [32], cucumber (*Cucumis sativus*) epidermis [33] and *Drosophila* imaginal discs [4].

Echinoids possess a hierarchically organized lightweight endoskeleton with a unique mechanical design [34–37]. Voronoi diagrams have been used to model echinoderm embryogenesis (e.g. starfish and echinoids) [38], as well as the pentamerism and skeletal growth of the echinoid test [39,40]. Recently, Yang *et al*. [41] investigated the three-dimensional microstructural design of spines in the sea urchin *Heterocentrotus mamillatus* describing it as Voronoi-like pattern but without providing a specific assessment. Hence, to our knowledge, Voronoi structures have been neither specifically assessed in echinoids nor described in detail in their tests at a microstructural level.

Numerous studies have been carried out on the echinoid endoskeleton architecture, with a particular focus on the variability in their highly porous microstructure and related mechanical properties [35,37,42–56]. The echinoid endoskeleton consists of stroma and stereom. The stroma includes sclerocytes, collagen fibrils and other extracellular matrix components, whereas the stereom primarily consists of magnesium-calcite, small amounts of stable amorphous calcium carbonate, water and intra-crystalline organic molecules [57–61]. The latter constitutes the intrastereomic organic matrix, which include different proteins, glycoproteins, and sulfated polysaccharides [57–61]. The stereom is organized in a three-dimensional mesh of trabeculae and its architecture is highly variable, reflecting its differentiated biological function [35,36,46,54,56,62,63]. During the 1970s, studies investigated the two-dimensional characteristics of the outer surface of the test plate stereom in some regular echinoid species [62,64]. Subsequently, Smith [46] systematically classified the different stereom types and their intra- and interspecific variability. Other studies underpinned a similar morpho-functional variability in the echinoid skeletal components, e.g. the specialized ossicles of the buccal apparatus [48]. Recently, studies analysed some echinoid stereom in three dimension providing internal insights of the trabecular system and mechanical behaviour [35,41,54,62,65]. In particular, the mechanical behaviour of the stereom structure is highly affected by the variability in material composition and porosity. For example, the Mg/Ca ratio content has been demonstrated to alter mechanical properties and is highly variable among different species, ossicles and stereom types as well as in association with different food type availability and environmental abiotic factors (e.g. temperature and salinity) [66–69]. The stereom porosity, mainly in spines, has also been investigated in detail [50,55,70–72]. Pore amount, size and variability are important microstructural features and often affect the physical properties of these materials such as elastic moduli, yield, rupture or ductile strength [51,73].

**Figure 1.**
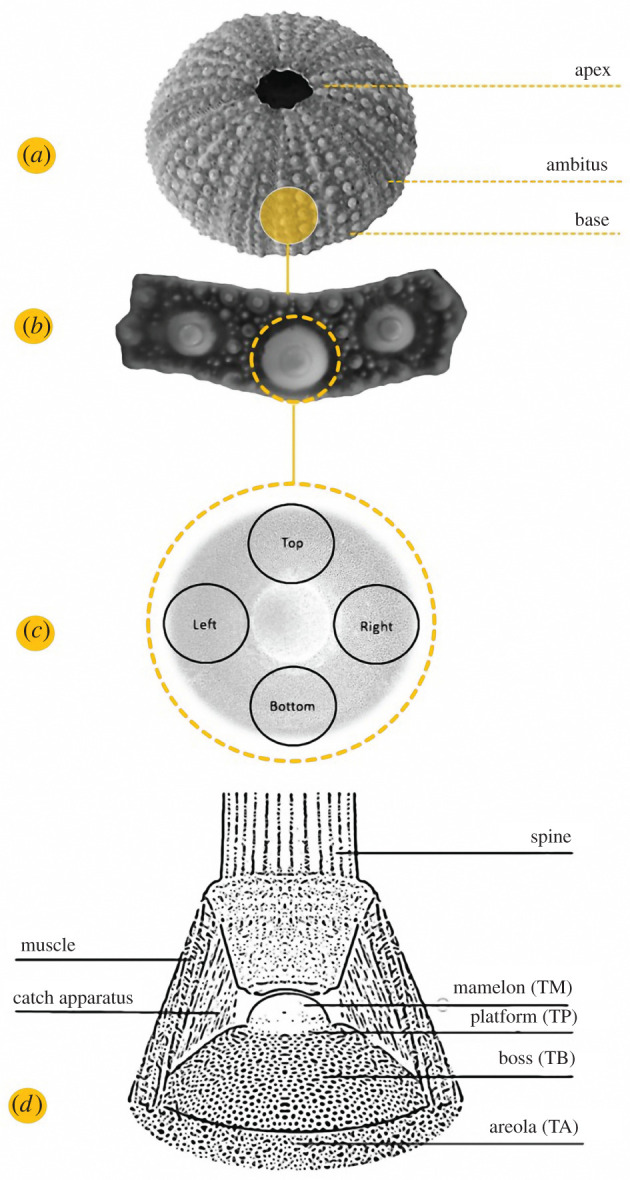
Echinoid test, skeletal plate, and tubercle zone. (*a*) *Paracentrotus lividus* test and (*b*) the extracted interambulacral plate. (*c*) Illustration of the four tubercle regions analysed: bottom, left, right and top. (*d*) Illustration of the primary spine tubercle and associated soft tissues (muscle and catch apparatus): (TM) mamelon, (TP) platform, (TB) boss and (TA) areola.

**Figure 2.**
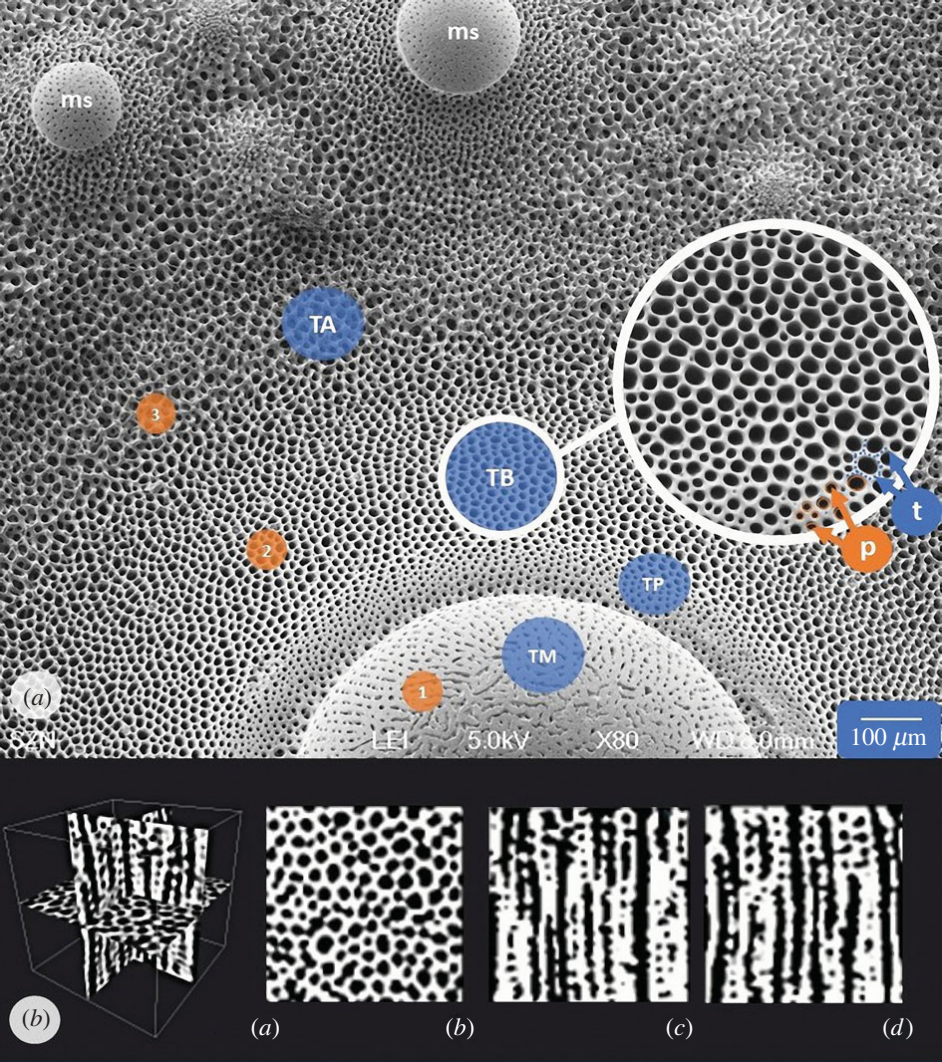
Tubercle architecture and regions examined. (*a*) SEM micrograph (top view) of *Paracentrotus lividus* interambulacral plate showing the primary spine tubercle and its stereom microstructural variability. Three stereom types can be recognized: (1) microperforate, (2) galleried, and (3) labyrinthic. In addition, the mamelon of secondary spines (ms) is also shown. The region topographic reference is underlined by a solid line circle in which the pores (p) and trabeculae (t) are indicated (arrows). (*b*) Micro-CT scan of tubercle boss subsection extracted by *P. lividus* interambulacral plate showing (*a*) transversal, (*b*) sagittal and (*c*) coronal views.

The aim of this study was to identify and geometrically describe the stereom skeletal pattern located at the tubercle boss of primary spines in the sea urchin *Paracentrotus lividus* (Lamarck, 1816). In particular, the tubercle is the spine attachment site and is morphologically variable consisting of: (1) mamelon, a dome-like solid structure of imperforate stereom functioning as a ball-and-socket joint where the spine is articulated; (2) smooth platform, located between the summit of the boss and the mamelon base, which allows a circular movement of the spine and consists of imperforated stereom; (3) boss, a conical dome-like lightweight construction characterized by galleried stereom, where a hollow truncated cone of collagenous ligaments, called catch apparatus, enters the pores; (4) areola, the area surrounding the base of the boss, consisting of fine labyrinthic stereom where longitudinal muscle fibres are attached [47,56,74–76] (figures 1 and 2).

The tubercle boss (TB) is a complex and mechanically stressed architecture that has attracted attention due to its rigorous microstructural regularity in contrast to the surrounding, less-organized trabecular mesh (figure 1). A rigorous analysis of the architecture of TB geometric patterns can improve the understanding of the mechanical function of the stereom, thereby widening the knowledge of the mechanical and geometrical characteristics of natural systems. In addition, the results can be used to inspire biomimetic technologies and designs. In this regard, the TB stereom was analysed by means of: (1) trabecular analysis, describing the TB stereom via segment-node configuration; (2) pore analysis, delineating the TB pore spaces and their centroids; (3) computational generation of the Voronoi model from pore space centroids and corresponding estimation with the trabecular system; and (4) characterization of the Voronoi pattern in the TB echinoid skeleton comprising an evaluation of the polygonal shapes, geometrical regularity and cell seed spatial distribution.

## 2. Material and methods

### 2.1. Material preparation and image acquisition

Specimens of *Paracentrotus lividus* (Lamarck, 1816) were collected by the scuba divers of the Stazione Zoologica Anton Dohrn in the Gulf of Naples, Italy (June 2019) from public and non-protected natural locations. Animal sampling was performed in full compliance with the European Union guidelines (European Directive 92/43/EEC of 21 May 1992 on the conservation of natural habitats and wild fauna and flora). The number of animals used for experimental purposes was reduced to minimum, and only samples required to obtain reliable data were used. Three tests (measuring respectively 4.9, 5.0, 5.2 cm in diameter and 2.9, 2.5, 2.9 cm in height) were digested in 0.1 N NaOH (for 1 week) to remove soft tissues. The samples were then washed three times in deionized water to remove caustic remains and air-dried for several days. Two types of interambulacral plates were extracted from the samples: (1) apical plates (*N* = 3), which represent the youngest plates in echinoid ontogeny and are small compared to older plates; and (2) ambital plates (*N* = 3), which represent the largest and most mature plates in echinoid ontogeny and are located at the ambitus (maximum horizontal circumference of the test). The plates were isolated, mounted on stubs and sputter-coated (Leica EM ACE 200) with a 10 nm platinum layer. Micrographs of the apical (*Ap*) and ambital (*Am*) TB stereom were recorded in four regions (bottom, left, right and top) (for a total of 24 micrographs) (figure 1), using a scanning electron microscope (SEM) (JEOL 6700F 250 MK2, at 5 kV, 150-fold magnification) at the Anton Dohrn Zoological Station in Naples, Italy (electronic supplementary material, figure S1a,b).

### 2.2. Image processing

Twenty-four SEM images of the TB stereom were recorded to a resolution of 410 × 1024 pixels. Micrographs were processed using Avizo software (v. 2019.1); Thermo Fisher Scientific, Waltham, MA, USA) and ImageJ Fiji software (v. 1.52e). The images were converted to binary images using an interactive manual thresholding to identify and separate the area of interest from the background. The binarized images were used for the skeleton analyses, pore analyses, assessment and geometrical characterization of the Voronoi pattern (electronic supplementary material, figure S1c,d,e).

### 2.3. Trabecular analysis

**Figure 3.**
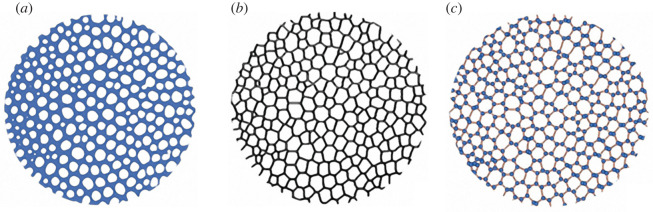
Trabecular analysis of *Paracentrotus lividus* TB stereom. (*a*) SEM micrograph binarization, (*b*) skeletonization of the binarized image, and (*c*) computation of segment-node configuration.

SEM images were skeletonized using the Avizo ‘auto skeleton’ command [77]. The skeletonization function transforms the TB stereom into a segment-node configuration: segments are lines equidistant to the boundaries of each trabeculae describing their shapes, and nodes are the junctions or intersection points of these segments describing their connectivity (figure 3). Node-segment configurations of the TB regions were analysed obtaining information regarding: (1) *nodes (n),* number and Euclidean coordinates (X,Y); (2) *segments (s),* number of segments; (3) *connectivity (C),* number of junctions per each node; (4) *chord length* (*l*_c_), length of the straight line between two nodes; (5) *curved length* (*l*_t_), length of the approximated curved course of a segment; (6) *tortuosity* (*t*), ratio between curved length and chord length *t* = *l*_t_/*l*_c_; (7) *radius (r),* mean value of all point radii describing a segment. Terminology follows Grun & Nebelsick and Avizo [35].

### 2.4. Pore analysis

**Figure 4.**
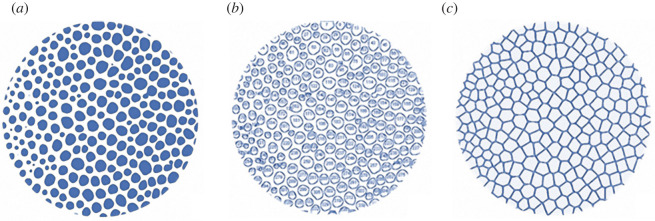
Pore analysis of *Paracentrotus lividus* TB stereom. (*a*) Stereom binarization, (*b*) pore identification and (*c*) computation of the Voronoi model from centroids.

The pore spaces were analysed from binarized SEM images of the TB regions using the ‘Analyze particles’ command in Fiji (figure 4). This provides information regarding: (1) *centroids* (*n_c_*)*,* centre points calculated as an average of the *x* and *y* coordinates of all the pixels in the selection; (2) *total area* (*A*_0_) of the selection in calibrated square units (μm^2^); (3) *pore area* (*A*_pore_) of the selection in calibrated square units (μm^2^); (4) *perimeter* (*Perim*), the length of the boundary of the selection calculated by its decomposition into individual selections; (5) *circularity* (*Circ*)*,* shape descriptor calculated using equation (2.1)

2.1 $$Circ=4\pi \times \left[{\left(\frac{A_{\mathrm{pore}}}{Perim}\right)}^{2}\right],$$

for which a value of 1 indicates a perfect circle and values decreasing toward 0 indicate increasingly elongated shapes; (6) *porosity* (*P*)*,* amount of pore area in the skeleton compared to the skeletal area investigated *in toto* (2).

2.2 $$P=\left(\frac{A_{\mathrm{pore}}}{A_{0}}\right)\times 100\text{\%}.$$

### 2.5. Voronoi model

A Voronoi model is a geometrical way of dividing space into a distinct number of regions or cells [7]. As stated in equation (2.3), beginning from a set of seeds φ = {*x*_1_, *x*_2_, … } specified in R*^d^*, a Voronoi cell C (*x_i_*_,_φ) of a point *x_i_*_,_ can be generated as the set containing all the points closer to *x_i_*_,_ than to any other seed [5].

2.3 $$C(x_{i,}\varphi )=\;\{y\in R^{d}\;:|\left|y-x_{i}\right||\leq |\left|y-x_{i}\right||\;\;\mathrm{for}\;\mathrm{all}\;\;j\neq i\}.$$

**Figure 5.**
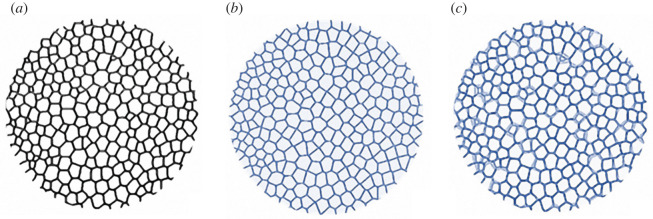
Voronoi divergence analysis of *Paracentrotus lividus* TB stereom. (*a*) Skeletonization of the binarized stereo; (*b*) computation of the Voronoi model; (*c*) superimposition of the actual stereom (*a*) and the Voronoi model (*c*).

Voronoi models were computed using the Delaunay Voronoi algorithm implemented in Fiji. From the centroids detected in the TB pore analysis (see ‘Pore analysis’), a series of lines equidistant to the two closest centroids were generated obtaining the borders of the Voronoi cell of each particle (figure 5). This procedure was applied using the Delaunay Voronoi dialog box in Fiji, which constructs Voronoi model images (*I*_v_) for each echinoid sample.

### 2.6. Divergence between skeletal pattern and Voronoi model

The TB skeleton (*I*_S_) and Voronoi (*I*_V_) images were compared using MATLAB R2020a (Mathworks, Inc., Natick, MA, USA). The images were translated into binary matrices (BWI_S_ and BWI_V_), and, subsequently, a matrix of differences as absolute value (*diff*) was calculated using equation (2.4). This matrix quantifies the differences between the two images where all the concordant pixels assume the value 0, while the discordant pixels assume value 1. The discordance between the *I*_S_ and *I*_V_ images (equation (2.5)) was calculated by the ratio between the number of discordant pixels (4) and the total number of pixels (*TP* = 410 × 1024).

2.4 $$diff=\mathrm{abs}({\mathrm{BWI}}_{S}-{\mathrm{BWI}}_{V})$$

and

2.5 $$\mathrm{disc}=\frac{\mathrm{diff}}{\mathrm{TP}}.$$

### 2.7. Tubercle boss Voronoi pattern characterization

**Figure 6.**
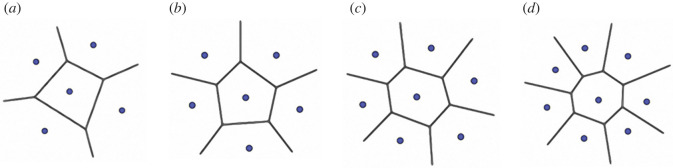
Voronoi polygonal shape. Planar Voronoi model generated from a seed having (*a*) four, (*b*) five, (*c*) six and (*d*) seven neighbour seeds.

Once the overlap between the TB skeletal pattern and Voronoi model was estimated, the geometrical properties of the echinoid TB Voronoi pattern were investigated defining the following parameters:

(1) *Number of seeds* (*n*_c_)*,* corresponding to the number of centroids (identified in ‘Pore analysis’).
(2) *Distance* (*d*) between two seeds, calculated using equation (2.6).
  2.6 $$d=\sqrt{{\left(Y_{2}-Y_{1}\right)}^{2}+{\left(X_{2}-X_{1}\right)}^{2}}$$
(3) *Number of neighbour seeds* (*n*_ns_)*,* the number of surrounding seeds computed for each seed as the number of cells sharing a boundary with the analysed seed cell. This was calculated *via* the nearest neighbour point analysis using R software (R core team, 2019) based on the centroid coordinates. The number of neighbour seeds and their distribution determines the number of cell sides (shape) in the Voronoi polygonal pattern (figure 6). It is worth noting that a tessellation composed of identical cells having six sides and vertex angles of 120° corresponds to a regular hexagonal honeycomb that is a fully ordered two-dimensional Voronoi model [5].
(4) *Regularity* (*α*), [12,13] in comparison with a regular hexagonal 2D Voronoi model, is estimated using equation (2.7):
  2.7 $$\alpha =\frac{\delta }{d_{0}}.$$
  Where *δ* is the minimum distance calculated on all the pairwise distances between seeds and *d*_0_ is their distance given by equation (2.8):
  2.8 $$d_{0}=\sqrt{\left(\frac{2A_{0}}{n_{c}\sqrt{3}}\right)},$$
  *A*_0_ is the total area and *n* represents the number of seeds.

A regular hexagonal honeycomb is characterized by *δ* = d_0_ and *α* = 1; whereas *δ* and *α* are close to 0 for a completely random (Poisson Voronoi) model [12,13].

(5) *Seed spatial distribution (ssd).* Statistical analyses based on Ripley's K-function [78] were carried out to characterize the seed spatial distribution of the echinoid TB Voronoi pattern. This function evaluates the distribution of the mapped two-dimensional spatial point data starting from the hypothesis of a *complete spatial randomness* (CSR) [79]. A map of points can be described as: (1) ‘random’ (CSR) if it is consistent with the null hypothesis of a binomial or homogeneous Poisson process; (2) ‘aggregated’ when the points are closely spaced in groups; (3) ‘regular’ when the points are more regularly spaced than expected under CSR [79]. In this framework, the K-function is defined by equation (2.9):
  2.9 $$K(h)=\lambda^{-1}E(h),$$

Where *λ* is the intensity of the point process of seeds, i.e. the mean number of points per unit area; *E*(*h*) denotes the expected number of further events within distance *h* of an arbitrary event. The K-function is usually estimated by equation (2.10):

2.10 $$K(h)=|A|{\left\{n(n-1)\right\}}^{-1}\overset{n}{\underset{i=1}{\sum }}\overset{n}{\underset{\;j=1,j\neq i}{\sum }}w_{ij}I(r_{ij}\leq h),$$

where |*A*| is the observation area, *n* the number of seeds, *r_ij_* the distance between the seeds *x_i_* and *x_j_*, *I* is the indicator function (i.e. *I*(*r_ij_* ≤ *h*) is equal to 1 if *r_ij_* ≤ *h* and is equal to 0 otherwise), and *w_ij_* are weights correcting the edge effect estimate (*w_ij_* is the proportion of the circumference of a circle centred at *x_i_* passing through *x_j_* contained in the observation region A).

The K-function of a homogeneous Poisson process is *K*(*h*) = *πh*^2^ and provides a benchmark against which it is possible to assess the pattern divergence from CSR. In aggregated patterns, *K*(*h*) tends to be larger than *πh*^2^ at distance *h* greater than the typical diameter of a cluster. Conversely, in regular patterns, *K*(*h*) is smaller than *πh*^2^ because each event tends to be surrounded by empty space [78,79]. The Min-Max credibility CSR band was obtained using the Monte Carlo method by simulating 20 random homogeneous Poisson point patterns with the same intensity of the considered one and estimating the *K*(*h*) function for each of them in different *h* values. The band was obtained by computing the minimum and maximum values of *K*(*h*) on the simulated patterns for each value of *h*. The variable *h* is varying in 512 equally spaced points of the (discretized) interval (0, H), where *H* is equal to ¼ of the minimum dimension of the window of observation. This choice is recommended [78,80,81] to reduce the bias of the estimate that may increase when *h* is large.

### 2.8. Statistical analysis

Multivariate approaches were used to compare the plate types and plate regions in terms of their geometric characteristics among the 24 micrographs analysed. All the variables included in the analysis were numerical interval variables. They included (1) number of nodes, (2) number of segments, (3) connectivity, (4) curved length, (5) chord length, (6) radius, (7) tortuosity, (8) total stereom area, (9) pore area, (10) pore perimeter, (11) pore circularity, (12) stereom porosity, (13) divergence estimation of Voronoi patterns, (14) number of Voronoi seeds, (15) distance between Voronoi seeds, (16) regularity of Voronoi elements and (17) number of neighbour seeds. Since the variables were expressed in multiple types of units of measurement, any variances were not meaningfully comparable across variables. Consequently, data were z-standardized prior to analyses. A permutation multivariate analysis of variance (PERMANOVA) [82] based on Euclidean distance was used for two fixed factors: (1) ‘plate type’, a fixed factor with two levels (apex and ambitus); and (2) ‘plate regions’, a fixed factor with four levels (bottom, left, right and top). Interactions between the two factors were also included in the PERMANOVA model. Estimates were based on 9999 random permutations [83]. Three separate PERMANOVAs with the same design were performed for each group of variables (i.e. trabecular analysis; pore analysis and TB Voronoi pattern characterization). Multivariate patterns were visualized using a non-metric multidimensional scaling (nMDS) based on Euclidean distances. All the analyses were computed in PAST software for Windows, v. 3.16. A significance level alpha = 0.05 was assumed for all the statistical tests.

## 3. Results

**Figure 7.**
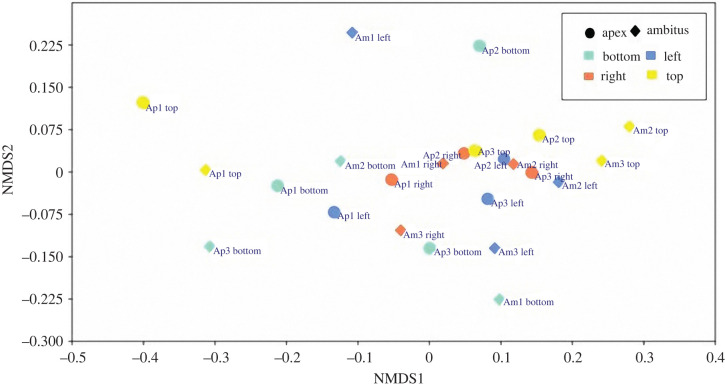
Non-metric multidimensional scaling (nMDS) ordination of apical and ambital samples for each plate region. Dimensions *k* = 2; stress = 0.12. ‘*Ap*’ and ‘*Am*’ represent the plate types and stand for apical and ambital plate, respectively; bottom, left, right and top represent the plate regions.

The PERMANOVA analyses show no statistically significant difference between plate types and plate regions (*N* = 24), and their interactions were also statistically insignificant (table 1). These results were consistent with the NMDS ordination (stress = 0.12, *k* = 2 dimensions), which showed an absence of groupings related to plate type or plate region (figure 7).

**Table 1.** Summary of two-ways PERMANOVA results. All the variables and different groups of variables (i.e. trabecular analysis, pore analysis, and tubercle boss (TB) Voronoi pattern) were considered. *N* = 24.

source of variability	d.f.	all variables			trabecular analyses			pore analyses			TB Voronoi pattern		
		MS	pseudo-F	*p*	MS	pseudo-F	*p*	MS	pseudo-F	*p*	MS	pseudo-F	*p*
plate region	3	16.044	0.840	0.578	53.601	0.578	0.794	6.178	1.156	0.327	4.837	0.864	0.547
plate type	1	52.111	0.273	0.952	33.379	0.360	0.819	12.534	0.234	0.869	0.623	0.111	0.984
interaction	2	10.698	0.560	0.872	54.268	0.585	0.792	3.212	0.601	0.759	3.447	0.616	0.773
residual	16	19.095			92.664			53.442			5.595		
total	23												

### 3.1. Trabecular analysis

In the TB apical plates, the analysed trabecular system is defined by 1522.92 ± 238.6 nodes (*N* = 18 275) and 2417.33 ± 504.4 segments (*N* = 29 008). Node connectivity is 3.0 ± 0.0 (*N* = 18 275), curved length is 9.33 ± 0.7 µm (*N* = 29 008) and chord length is 9.05 ± 0.7 µm (*N* = 29 008). Segment tortuosity *τ* is 1.02 ± 0.0 (*N* = 29 008) with a radius of 2.55 ± 0.2 µm (*N* = 29 008) (table 1; electronic supplementary material, S2 table).

In the TB ambital plates, the trabecular system consists of 1552.83 ± 299.3 nodes (*N* = 18 634) and 2321.08 ± 465.3 segments (*N* = 27 853). Node connectivity is 3.0 ± 0.0 (*N* = 18 634), the curved length is 9.58 ± 0.8 µm (*N* = 27 853) and the chord length is 9.30 ± 0.8 µm (*N* = 27 853). Segment tortuosity *τ* is 1.02 ± 0.0 (*N* = 27 853) with a radius of 2.65 ± 0.7 µm (*N* = 27 853) (table 1; electronic supplementary material, S2 table).

### 3.2. Pore analysis

In the TB apical plates, the pore area is 87.08 ± 25.0 µm^2^ (*N* = 10 241) with a perimeter of 34.30 ± 5.9 µm (*N* = 10 241), porosity is 43.02 ± 7.2% (*N* = 10 241) ranging from 26 to 55% and circularity is 85.76 ± 3.1% (*N* = 10 241) (table 2; electronic supplementary material, S3 table).

**Table 2.** Summary of trabecular and pore analysis results.

	trabecular analysis							pore analysis			
	*n*	*S*	*C*	*lt*	*l*c	*t*	*r*	*Apore*	*Perim*	*Circ*	*p*
*Ap*	1522.92 ± 238.6	2417.33 ± 504.4	3.0 ± 0.0	9.33 ± 0.7	9.05 ± 0.7	1.02 ± 0.0	2.55 ± 0.2	87.08 ± 25.0	34.30 ± 5.9	85.76 ± 3.1	43.02 ± 7.2
*Am*	1552.83 ± 299.3	2321.08 ± 465.3	3.0 ± 0.0	9.58 ± 0.8	9.30 ± 0.8	1.02 ± 0.0	2.65 ± 0.7	92.37 ± 31.6	35.05 ± 6.7	85.0 ± 3.0	44.10 ± 8.5

In the TB ambital plates, the pore area is 92.37 ± 31.6 µm^2^ (*N* = 10 180) with a perimeter of 35.05 ± 6.7 µm (*N* = 10 180), porosity is 44.10 ± 8.5% (*N* = 10 180) ranging from 31 to 57% and circularity is 85.0 ± 3.0% (*N* = 10 180) (table 2; electronic supplementary material, S3 table).

### 3.3. Divergence between skeletal pattern and Voronoi model

**Figure 8.**
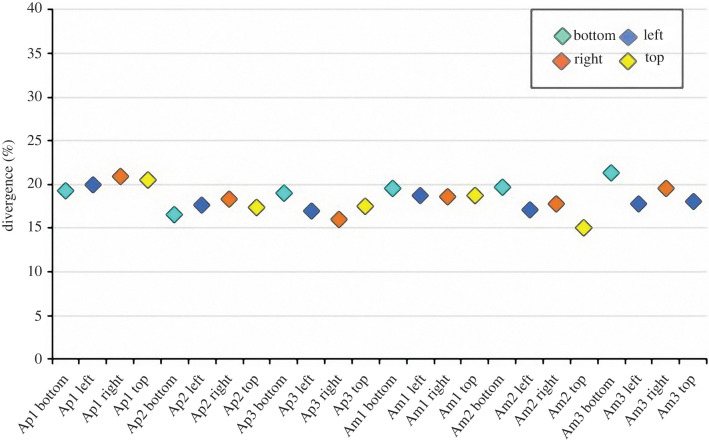
Divergence trend of the skeleton and Voronoi comparisons. ‘*Ap*’ and ‘*Am*’ represent the plate types and stand for apical and ambital plate, respectively; bottom, left, right and top represent the plate regions.

The matrix comparisons between the TB skeleton (*I*_s_) and Voronoi (*I*_V_) images indicated a divergence between these two tessellations of 18.0 ± 1.5% resulting in a concordance of 82.0 ± 1.5% (figure 8). The results are remarkably consistent in all the analysed plates (figure 8) (electronic supplementary material, S4 table).

### 3.4. Tubercle boss Voronoi pattern characterization

In the TB apical plates, the number of seeds is 853.42 ± 183.8 (*N* = 10 241), their distance is 90.54 ± 4.0 (*N* = 10 241) and regularity (*α*) is 0.47 ± 0.0 (*N* = 10 241) (electronic supplementary material, S5 table). In the TB ambital plates, the number of seeds is 848.33 ± 212.8 (*N* = 10 180), their distance is 91.06 ± 3.7 (*N* = 10 180) and regularity (*α*) is 0.45 ± 0.0 (*N* = 10 180) (electronic supplementary material, S5 table).

**Figure 9.**
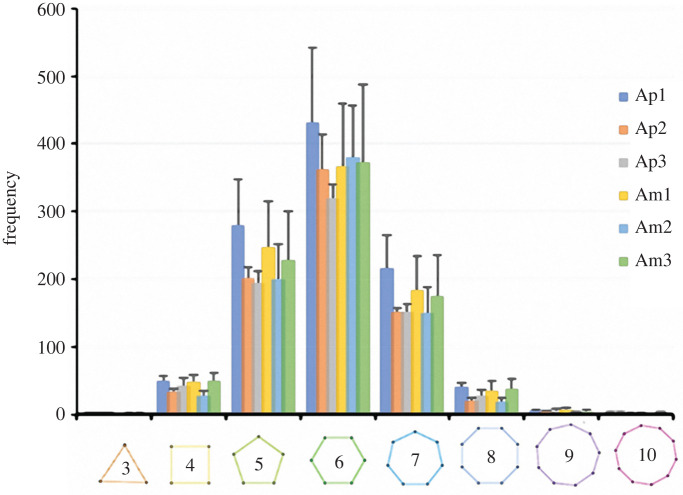
Histograms of number of neighbour seeds or number of TB Voronoi cell sides. Mean values (±SD) for each plate type sample (*Ap* = apical plate and *Am* = ambital plate) among its regions (bottom, left, right and top).

The number of neighbor seeds is 6.0 ± 0.0 (*N* = 20 421) ranging from 2 to 15 (*N* = 20 421). The neighbour data also correspond to the distribution of polygons constituting the TB Voronoi pattern in echinoids according to their shape. Thus, the median TB Voronoi cell sides conform with a constant equilibrium distribution of hexagons (44%), followed by pentagons (26%), heptagons (20%), quadrilaterals (5%), octagons (4%) and nonagons (1%) (figure 9).

**Figure 10.**
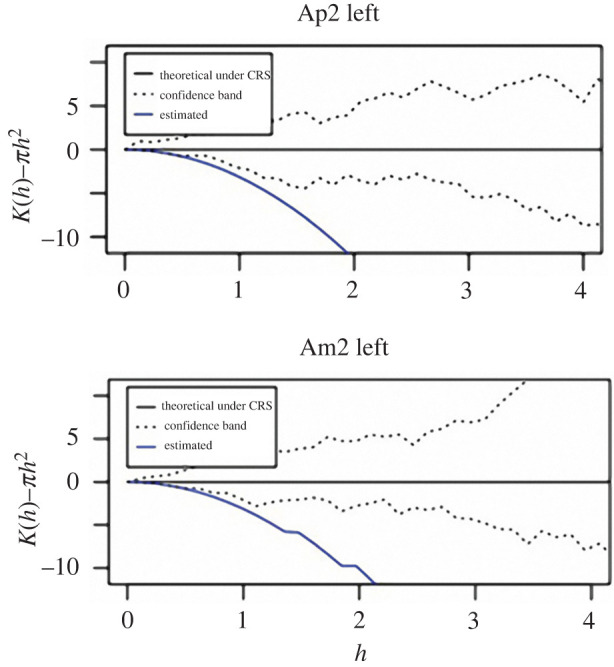
*K* function estimation results. The blue line represents the estimation of *K*(*h*)−πh^2^ for apical (Ap2 left) and ambital (Am2 left) plates. The plotted functions (blue lines) indicate that *K*(*h*), varying *h*, is smaller than πh^2^ and lies below the confidence band (dashed lines) departing from CSR.

The estimated *K*(*h*)-*πh*^2^ for TB stereom at apical and ambital plates was always located below the confidence band for CSR, obtained through a Monte Carlo simulation. The functions (blue lines) indicate that *K*(*h*), varying *h,* is smaller than *πh*^2^ and lies below the confidence band (dashed lines) predicted for CSR (figure 10). In the case of CSR, the estimate function should approximate the horizontal line located within the dashed confidence band. The fact that the estimate functions are below the CSR confidence region indicates a regularly spaced seed distribution for all the analysed samples.

## 4. Discussion

The TB stereom microstructure of *P. lividus* was analysed and geometrically described, leading to the first identification of a hexagonal and regularly spaced Voronoi pattern in the echinoid skeleton.

The image divergence estimation between the TB trabecular system and the generated Voronoi model revealed that the TB stereom geometrically diverges from a predicted Voronoi model by only 18%. Deviations from a computational generated Voronoi pattern resulting less than 20% can be interpreted as acceptable since naturally grown structures are subject to constraints such as genetic heritage and phenotypic plasticity. In fact, numerous patterns in nature, both in the plant and animal kingdom, such as coenobial green alga (*Prediastrum boryanum*), retinal pigment and lung epithelial cells of chick embryo and blastular cell sheet of starfish (*Asterina pectinifera*) are typically described as Voronoi Dirichlet polygonal constructions, although they present deviations from the mathematical models [32,84].

The TB Voronoi characterization indicated that the polygonal cell shapes describing the echinoid pattern are predominately hexagons (44%) (figure 8). In nature, hexagonal cells are common (e.g. beeswax honeycombs and cell wall of wood composed of cellulose, hemicelluloses and lignin). These structures provide functional mechanical properties that inspired novel designs of high-performance man-made structures, from macroscale architecture and engineering, such as the Hex tower architecture by Michel Rojkind, construction panels and bricks, turbine seal and mirror for Hubble space telescope, to micro- and nanofabrication, such as lithiated silicon, polyethylene crystalline fibres, ice-template-induced scaffold and anthraquinone molecules [31,40]. A regular geometric array (grid) constituted by simple polygons able to tile a two-dimensional or three-dimensional plane without any overlaps and gaps can assume only one of three forms: triangular, quadrilateral or hexagonal [85]. Hexagons divide a dimensional space using the smallest perimeter with non-aligned edges having trivalent vertices [6,15,31,85]. In the *P. lividus* TB regions, the prevalence of hexagons can therefore result in an optimally packed array, minimizing the amount of secreted calcite for the skeleton construction [1]. Moreover, unalienated trabeculae arranged in trivalent vertices (Y-shaped) within the plane can provide structural stability [35,86,87]. In particular, stresses within one of three converging trabeculae are redirected in two different directions. Furthermore, the axial stiffness of each trabecula converging to a vertex contributes to the global stiffness of the vertex along unalienated directions. These results are coherent with the visual identification of the approximated hexagonally arranged TB channels of the echinoid *Eucidaris tribuloides* detected by Smith *et al*. [56]. Nonetheless, in addition to hexagons, the TB Voronoi pattern is also described by an approximated equal number of pentagons (26%), heptagons (20%) and lesser frequencies of other polygonal types, which is a stable equilibrium distribution of polygons previously detected in both animal and plant tissues: e.g. chick embryo retinal pigment cells, lung epithelial cells [32] and cucumber epidermis [33]. Recently, this equilibrium has also been mathematically modelled and empirically confirmed in *Drosophila* wing discs, *Xenopus* tail epidermis and *Hydra* epidermis [4]. Gibson *et al*. [4] demonstrated that this distribution of polygonal cell types is a direct mathematical consequence of cell proliferation and could underlie developmental constraints related to growth, pattern formation and morphogenesis throughout metazoans. The echinoid TB stereom displays a similar equilibrium topology in both early forming apical and mature ambital plates. Nevertheless, the nature of the stereom and its structural growth mechanisms are completely different from the growing tissue of proliferating cells. Indeed, the echinoid skeleton is produced by intercellular calcite deposition within a syncytium of sclerocytes, i.e. a multinucleate cell formed by multiple sclerocyte fusion [61,88,89,90–93]. Syncytial sclerocytes generate a continuous cytoplasmic sheath surrounding a spacious vacuolar cavity, where the mineral lies and is coated with an organic matrix (trabecle coat) [88,89]. The synplasmic sheath and the matrix secrete and coat the calcareous ossicle trabeculae [88,89]. The morphogenesis is similar in all the stereom types: initial small, conical projections are generated, and then lateral bridges are formed resulting in a fine stereomic mesh, which is gradually thickened by secondary deposition of mineral [94–96]. Hence, a possible meaning of this common polygonal topology may be related to an optimally packed array for these convex structures in which polygons should cover a space plane without any overlaps or gaps [1,84].

A polygonal construction such as the Voronoi meshwork can vary from highly irregular to fully ordered, based on seed distance and disposition [12,13]. In the echinoid TB trabecular system, the parameter used to quantify the regularity of a two-dimensional Voronoi pattern was 0.5 ± 0. This indicates that the echinoid TB Voronoi pattern possesses a geometric order in comparison to a fully ordered regular hexagonal honeycomb. The divergence is a consequence of the TB centroid/seed arrangement, which also determines the specific polygonal topology that is not characterized only by hexagons. However, the significant regularity of this spatial seed organization was also underlined by Ripley's K function estimation. These findings suggest that the echinoid TB stereom structures approximates a regularly spaced Voronoi model, which can provide new insights regarding structural mechanics and growth principles in *P. livius* plates.

Numerous studies have dealt with two-dimensional and three-dimensional Voronoi constructions and their mechanical performance [9–15,26]. In this regard, Young's moduli and Poisson's ratio seem to be highly influenced by cell density and shape [9,14–16]. Random Voronoi constructions are more sensitive to fatigue than a hexagonal array, indicating that regular geometrical structures are the most resistant [9]. Cell wall defects induce weakness as demonstrated by various numerical analyses [9–15]. Thus, in the TB region of the echinoid test, the constructional solution to adopt a microstructural galleried pattern that approximates a hexagonal regularly spaced Voronoi construction represents a strategy to enhance local mechanical strength. This strength is particularly critical for TB regions subject to the unique action of spine ligaments consisting of cylindrical, parallel and regularly organized bundles, which uniformly insert into the stereom pores to depths of more than 100 µm [46,47,56,74–76,97]. It can be therefore hypothesized that this Voronoi organization is also reflected in this unique collagen fibre bundle disposition. Since these ligaments consist of mutable collagenous tissues, their mechanical properties can vary in tensile strength and stiffness through a direct control of the nervous system in coordination with the contractile activity of the spine muscles [46,47,98–101]. These muscles run longitudinally from the base of the spine to the areola region and, when contracted, the spine can freely move in any direction around the ball-and-socket joint. In this state, the catch apparatus ligaments are in a de-stiffened condition and the TB stereom is subject to very low levels of compressive and tensile stresses. However, the spine can be rapidly locked in a rigid position by the stiffening of the spine ligaments as a defense strategy from predators and wave action [56,74–76,97,98]. In this latter state, the TB stereom is exposed to high tensile stress, especially if the spine is also loaded by external forces, e.g. due to predator attack [102]. The ligaments can resist these forces and transfer directional stresses to the TB stereom. In this condition, the ligament can have a tensile strength of up to 40 MPa, which is within the range reported for mammalian tendons [76,101,103]. Plausibly, this unique behaviour of the catch apparatus system could explain the need to maximize the structural strength at the TB stereom using a directional and regular Voronoi construction with respect to the surrounding labyrinthic areola regions adapted to standard stresses. Indeed, Voronoi and hexagonal structures exhibit isotropic properties and very limited post-yield softening, making them ideal candidates for energy mitigation applications [15]. The areola region is conversely associated with different soft tissues and related mechanical needs [47,56,89,98]. Muscles are inserted in the less organized labyrinthic stereom of the areola and are indirectly joined to the skeleton by means of composite tendon-like structures, which consist of unstriated tendons and bundles of collagen fibrils, coiling around the stereom trabeculae. This area is exposed to multidirectional stresses related to muscle action and a freely movable spine [35,47,89,98]. The stereom variability also supposedly reflects different growth rates [46]. Smith [46] reported that a labyrinthic stereom is associated with a moderate or fast stereom growth, whereas the more regular stereom pattern (e.g. galleried) is related to a moderate growth rate that maintains pore alignment. Recently, Gorzelak *et al*. [96] reported an opposite pathway, i.e. denser stereom microfabrics (perforate, imperforate or labyrinthic stereom) generated more slowly than a regular galleried stereom, which could be rapidly produced. In this complex framework, Voronoi models result from specific growth processes and are therefore used to model growth patterns [5]. Euclidean Voronoi models correspond to constant speed growth (e.g. giraffe fur and turtle shell) [5,104]. Thus, a good approximation to the regular Voronoi model further suggests that the structural growth processes of the echinoid TB skeletal structure could correspond to a uniform synchronization in trabeculae growth rates.

The results achieved showed that TB trabeculae are uniformly long and possess little tortuosity, indicating that the trabeculae follow a direct course between two nodes. Similar results have been reported for the dwarf sea urchin *Echinocyamus pusillus* [35]. Linear trabeculae possess an increased flexural stiffness [35] and strength that are important in the boss region of tubercles exposed to high stress loads induced by spine movement, locomotion, or anti-predatory defense ([34] and literature cited therein).

The pore analysis provided information on the TB pore area and its geometry. Porosity is around 44% on average, in accordance with the plate porosity of 30 to 50% reported in literature [44,105]. The circularity values (*Circ*) were close to 1, indicating that pore shapes approximate circles. Porosity is usually highly variable in the skeletal components of echinoids, e.g. ossicles of the buccal apparatus and spines [48,54,55,106]. Variation in porosity creates distinct structural units contributing to the mechanical strength of the skeleton. Regularly ordered pore structures reported here represent structural solutions known to be mechanically efficient when loads are applied from predictable directions [107]. Recently, these structures have received increasing attention due to their potential application in the fields of electronics, photonics, and material sciences [106–109]. In the case of the sea urchin, TB region, loads and resultant stresses are typically predictable since they are correlated with regularly organized collagenous fibers that penetrate the pore space and spine locking mechanism [46,47,56,74–76,97,98].

In conclusion, this study provides critical analyses on the structure and design of the echinoid *P. lividus.* The microstructure of the echinoid stereom was demonstrated to be consistent with a Voronoi construction, providing a new perspective for investigating and modelling the different echinoid stereom structures, soft tissues disposition, mechanical skeletal adaptations, and structural growth mechanisms at two-dimensional and three-dimensional scale. Further quantitative documentation of microstructural patterns demonstrated by echinoids can also inspire and motivate the design implementation of various load carrying rod systems, in which lightweight and structural robustness are of prime importance. The echinoid stereom may be of broad biomimetic relevance to many applied fields, including engineering, architecture, biotechnology, and material sciences.

## Data Availability

All data are provided in electronic supplementary material [110].
